# Comparison between Subjective Sensations during First and Second Phacoemulsification Eye Surgeries in Patients with Bilateral Cataract

**DOI:** 10.1155/2016/6521567

**Published:** 2016-04-27

**Authors:** Ji-guo Yu, Ting Ye, Qing Huang, Yi-fan Feng, Jue Wang, Xun-an Fu, Yi Xiang

**Affiliations:** ^1^Department of Ophthalmology, The Central Hospital of Wuhan, Wuhan, Hubei 430014, China; ^2^Department of Ophthalmology, The Puai Hospital of Wuhan, Wuhan, Hubei 430033, China; ^3^Department of Ophthalmology, Zhongshan Hospital, Fudan University, Shanghai 200032, China

## Abstract

*Purpose.* To evaluate and compare the subjective sensations reported by patients during first and second cataract extractions.* Methods.* Consecutive patients undergoing bilateral sequential cataract extraction using phacoemulsification were recruited. Following cataract surgery, patients completed questionnaires designed to evaluate subjective sensations, including anxiety, eye bulges, pain, and light sensitivity. Changes in painful sensations experienced by patients between the two surgeries were also recorded. Comparisons were also performed for each subjective sensation between different age groups (<50, 50–59, 60–69, 70–79, and >79 years).* Results.* A total of 127 patients were included in the final evaluation. Statistical comparison of the results showed that there were significant differences in perception of anxiety, eye bulges, and pain scores between the first and second cataract surgeries (*P* < 0.05). However, there was no statistically significant difference for light sensitivity scores between the two surgeries (*P* = 0.555). The differences in anxiety, perception of eye bulges, pain, and light sensitivity scores between both the surgeries showed no correlation with age (*P* > 0.05 for all).* Conclusions.* Our research confirms the common observation that patients with bilateral cataracts often report more ocular discomfort during the second surgery. There are, therefore, additional factors that should be considered upon treating patients with bilateral cataracts, and the provision of preoperative counseling could play an important role in providing adequate patient care.

## 1. Introduction 

Phacoemulsification cataract surgery is the most commonly used and effective surgical method for the treatment of cataract to improve a patient's visual function and quality of life [1–3]. Shorter operative times, smaller incisions, and immediate visual recovery are all benefits of this treatment. Although it can reduce the economic burden, simultaneous surgery for patients with bilateral cataracts may cause serious bilateral complications, such as endophthalmitis [4]. Therefore, for these patients, consecutive surgery is the preferred approach for treating both eyes.

Subjective sensations, such as ocular pain and light sensitivity, are known to occur during phacoemulsification surgical procedures [5, 6]. It has been reported that there is a difference in bilateral cataract patients' assessment of subjective sensations between the first and second cataract surgeries [7, 8]. Intraoperative ocular pain has been investigated rather extensively; however, there are fewer studies regarding the differences in pain perception between the first and the second cataract surgery. Some studies have found that patients report more pain during the second cataract surgery than during the first [9, 10], whereas others found no correlation between the level of pain perception and the sequence of operations [11–13]. However, pain is only one of several sensations that have been reported by patients who have undergone this surgery, and other sensations such as eye bulges, anxiety, and microscope light sensitivity have been noted. Eye bulges reflect the sensitivity to intraocular perfusion pressure, which have not reached the degree of pain. Fewer reported pieces of data are currently available regarding these sensations, and the correlation between these sensations and age has yet to be investigated. Therefore, the aim of our study was to compare bilateral cataract patients' evaluations of subjective sensations, including intraoperative anxiety, eye bulges, perceptions of pain, and light sensitivity, between first and second phacoemulsification surgeries.

## 2. Materials and Methods

This study was approved by the institutional ethics committee of the Central Hospital of Wuhan and followed the tenets of the Declaration of Helsinki. Patients affected by bilateral cataracts were eligible for this study. Patients were enrolled if they underwent phacoemulsification cataract surgery at the Central Hospital of Wuhan (Hubei, China) between January 2015 and September 2015. The first surgery was performed in the eye with a higher-grade cataract or otherwise poorer vision. The other eye was operated on within approximately 1 month, based on the patient's choice. All patients who had uneventful phacoemulsification under topical anesthesia using proparacaine 0.5% (Alcaine®) with the placement of an intraocular lens in the capsular bag were included. A preoperative mydriatic was administered, and the pupil size remained at more than 6 mm throughout the operation. All surgeries were performed under the same conditions. The exclusion criteria were cataract with glaucoma, high intraocular pressure, lens dislocation, and posterior capsular rupture during surgery that prolonged the procedure by 10 min or more. The procedures were fully explained to each patient, and each patient provided written informed consent.

All procedures were performed by a single surgeon using the same phaco machine (Stellaris PC, Bausch & Lomb) between 9:00 and 12:00 am. The surgical technique was clear corneal phacoemulsification through a 3.2 mm incision located between sectors 10 and 12. In all cases, foldable intraocular lenses were implanted using the dedicated injector. Only sutureless cases were included in the final evaluation. The operation time was recorded by an assistant doctor, as the time from the opening of the side port incision to the removal of the lid speculum. After the cataract surgery, patient questionnaires were used to evaluate subjective sensations. Every patient was accompanied to the recovery room and asked to grade the pain they experienced during the procedure using the Numerical Rating Scale (NRS) with scores from 0 to 10, where 0 means no pain, 1–3 mean mild pain, 4–6 mean moderate pain, and 7–10 mean severe pain [14, 15]. Anxiety scores were graded using the Amsterdam preoperative anxiety and information scale (APAIS) [16]. Eye bulges scores reflected the intraocular perfusion pressure (0 = no, 1 = minimal, 2 = mild, 3 = moderate, and 4 = severe), and microscope light sensitivity was measured and scored (0 = no, 1 = minimal, 2 = mild, 3 = moderate, and 4 = severe) [17]. Patients were instructed to select a single number that best represented the level of each subjective sensation that they were experiencing. Over a period of 1 month following the procedure, cataract surgery was performed on the remaining affected eye, after which patients were asked to complete the same questionnaire, with additional questions comparing the pain experienced during each surgery.

The data was collected using a structured study form and a questionnaire. The following patient data was collected and analyzed: patient age and sex, concomitant diseases, operative time, anxiety score, eye bulges score, pain score, and light sensitivity score. For each score, subgroups were also assigned and compared based on patient age (<50, 50–59, 60–69, 70–79, and >79 years).

All data were recorded in Microsoft Excel spreadsheets and analyzed using the Kolmogorov-Smirnov test for normal distribution. Continuous variables were expressed as the mean ± standard deviation for those displaying normal distribution or otherwise using the median. A Mann-Whitney *U* test was used to estimate the differences in each score between the first and second surgery. For the comparison of parameters in different age subgroups, a chi square test with a 5 × 4 contingency table was used. Correlation between subjective sensation and age was assessed using Spearman's correlation test. Statistical analysis was performed using SPSS PASW Statistics version 18.0 software (IBM Corporation, Armonk, NY, USA). *P* values less than 0.05 were considered statistically significant.

## 3. Results

A total of 132 consecutive patients were enrolled in this study. However, 5 patients gave up treatment before undergoing the second surgery. Hence, 127 patients (60 men and 67 women) aged between 43 and 93 years (median 70 years) were included in the final evaluation. Patient demographic characteristics and concomitant diseases are recorded in Table 1.

**Figure 1:**
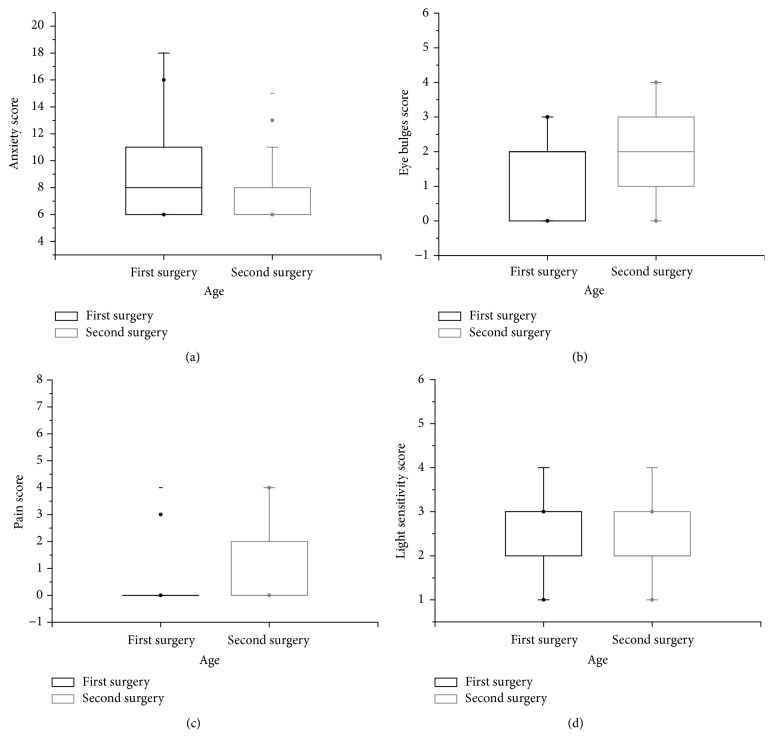
Comparison of anxiety, eye bulges, pain, and light sensitivity scores between first and second phacoemulsification cataract surgery. The bar in the box indicates the median. The lower and upper hinges indicate the interquartile range (IQR). The whisker extends to the most extreme data point that is no more than 1.5 times the IQR. Dots outside the box represent values outside fences (outliers). (a) Anxiety score. (b) Eye bulges score. (c) Pain score. (d) Light sensitivity score.

The average operation time was 11.5 ± 1.9 min for the first surgery and 11.7 ± 1.8 min for the second surgery. The difference between operation times was not statistically significant (*P* = 0.317). All the scores that were obtained did not follow a normal distribution (*P* < 0.05, Kolmogorov-Smirnov test); therefore a Mann-Whitney *U* test was used to estimate differences of these parameters between the first and second surgery. The comparative analysis of anxiety, eye bulges, pain, and light sensitivity scores between the first and second phacoemulsification cataract surgeries is shown in Figure 1. The results from statistical analyses show that there was a significant difference in the anxiety, eye bulges, and pain scores between the first and second surgeries (*P* = 0.001, *P* = 0.049, and *P* = 0.011, resp.). Patients experienced less anxiety, a greater number of eye bulges, and pain during the second cataract surgery compared to the first surgery. However, differences in the scores for light sensitivity between surgeries were not statistically significant (*P* = 0.555).

**Figure 2:**
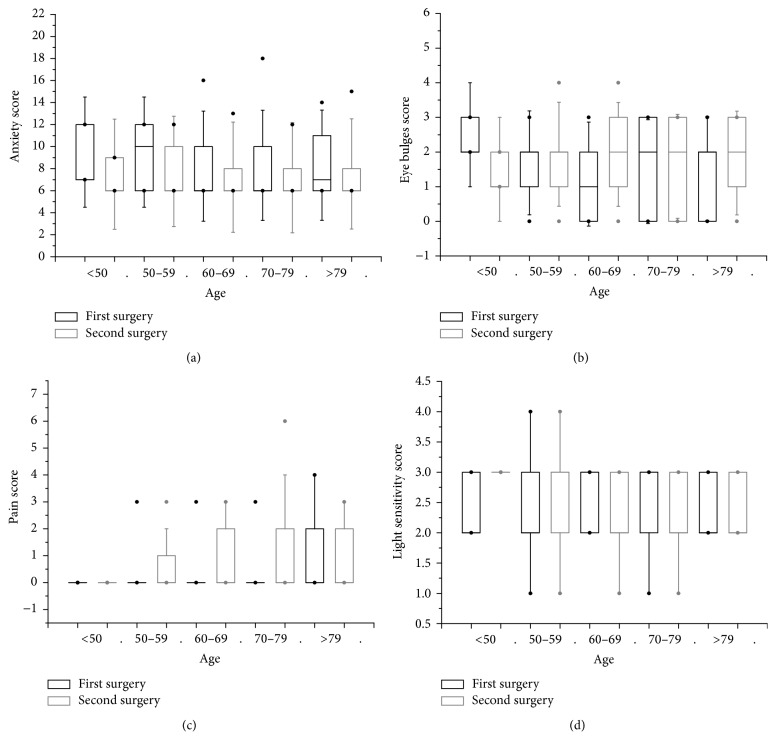
Comparison of anxiety, eye bulges, pain, and light sensitivity scores for different age groups (<50, 50–59, 60–69, 70–79, and >79 years). The bar in the box indicates the median. The lower and upper hinge indicate the interquartile range (IQR). The whisker extends to the most extreme data point that is no more than 1.5 times the IQR. Dots outside the box represent values outside fences (outliers). (a) Anxiety score. (b) Eye bulges score. (c) Pain score. (d) Light sensitivity score.

The comparative data for the different age subgroups (<50, 50–59, 60–69, 70–79, and >79 years) for anxiety, eye bulges, pain, and light sensitivity score are shown in Figure 2. Statistical analysis demonstrated no significant differences in anxiety, eye bulges, pain, and light sensitivity scores between the first and second surgery among the different age groups (*P* = 0.062, *P* = 0.379, *P* = 0.962, and *P* = 0.122, resp.). Additionally, the differences in anxiety, eye bulges, pain, and light sensitivity scores between the first and second surgery had no correlation with age (*r*_anxiety_ = −0.052, *P* = 0.564; *r*_bulges_ = 0.029, *P* = 0.748; *r*_pain_ = −0.088, *P* = 0.328; and *r*_light_ = −0.137, *P* = 0.123, resp.).

Our analysis shows that 33 (26.0%) patients reported more pain during the first cataract surgery compared to the second surgery. Fifty-three (41.7%) patients reported more pain during the second cataract surgery than during the first surgery, while 35 (27.6%) patients reported the same pain score during both surgeries. Six (4.7%) patients reported no pain during both the first and the second surgeries. The numbers of patients in the different age groups who reported changes in painful sensation score between the first and second surgeries are presented in Table 2.

## 4. Discussion 

In clinical practice, it is a common observation among ophthalmic surgeons that patients with bilateral cataract often report more pain and discomfort during the second consecutive eye surgery. Previous studies have investigated this observation and have also reported an increase in pain in the second surgery relative to the first [4, 9, 10]. In agreement with their data, our results confirm that the majority of patients do in fact experience more pain during the second cataract surgery compared to the first. However, in our study, 33 patients reported more pain during the first surgery, 35 patients reported the same pain score for both operations, and 6 patients reported no pain for either. Therefore, an increase in pain experienced from the first to the second surgery is not consistent for all individuals.

Eye bulges, which reflect sensitivity to intraocular perfusion pressure, were also found to increase in severity following the second cataract extraction. There are most likely multiple factors that contribute to the sensitivity of the eye to perfusion pressure. We propose that this observation might be related to factors such as the lens nuclear hardness, concomitant diseases, type of lens opacity, inflammatory cytokines, and psychological factors. These may be the same factors that contribute to ocular pain. Our study found no correlation between patient age and differences in eye bulges and pain score between the first and second surgery, which implies that intraoperative perception of discomfort is unaffected by age.

It is well known that psychological factors, such as mental stress, are present beforehand and influence patient responses to cataract surgery. The degree of mental stress most likely contributes to the degree of pain reported by patients. It is also likely that patients often have more mental stress before their first cataract surgery [18]. Additionally, the eye with a higher cataract grade is normally operated on first, so patients can have higher expectations of success from the first procedure. A resulting increase in positive thinking, as well as therefore increased cooperation with doctors, might positively influence patient pain thresholds. We suggest that these factors could diminish the pain patients experience during the first surgery [8]. However, previous studies have found that patients were more relaxed for their second-eye surgery, which is consistent with our findings and explains why patients experienced increased pain during the second procedure [19, 20]. Additionally, patients know what to expect from the second operation, therefore preoperative anxiety is decreased, and decreased anxiety may lead to increased awareness during the procedure, making the sensitivity to pain increased, and result in a negative experience during the second surgery [8]. Alternatively, a pharmacological explanation for this observation is that previous exposure to anesthesia medication during the first procedure leads to drug tolerance, so that the response to the same medication is diminished during the second procedure [9].

We have discussed the role of subjective psychological factors in patients' perception of pain during second cataract surgeries. However, objective molecular mechanisms, for example, sympathetic irritation in the second eye resulting from surgery in the first, might also contribute to this phenomenon. Zhu et al. reported that expression of MCP-1, a pain-related inflammatory chemokine, is significantly increased in the aqueous humor of the contralateral eye following first cataract surgeries. This suggests that there may be a sympathetic ophthalmic type uveitis in the contralateral eye after surgery, which may explain why phacoemulsification of the second eye is more painful [21]. Not all patients reported more pain for the second surgery: some reported more pain during the first surgery, whereas others reported either the same or no pain for both procedures. This implies that the sensitivity of patients to inflammatory factors may vary.

Patients undergoing cataract surgery can experience a wide range of subjective sensations. Light sensitivity is one example. Some studies have also reported that patients can see instruments and movement within their visual field during surgery, and in some cases, where vision is lost completely during surgery, light sensitivity was still present for each patient [22–24]. High light sensitivity accompanying photophobia symptoms following cataract surgery can result in increased psychological distress for some patients [17]. This could in turn influence their intraoperative pain perception. However, our analysis found that the differences in light sensitivity score between the two surgeries were not statistically significant. Different methods of anesthesia can affect the severity of light sensitivity [17], and the conditions for use of topical anesthesia for the patients included in our study were identical. We suggest that this is why the light sensitivity scores for the two operations were not significantly different. We therefore postulate that light sensitivity does not play an important role in either eye bulges scoring or pain perception between the two cataract surgeries.

In conclusion, our research confirms the common observation that patients with bilateral cataract often experience more pain or ocular discomfort during the second consecutive cataract surgery, compared to the first procedure. The differences in subjective sensations, such as anxiety, eye bulges, ocular pain, and light sensitivity, between the first and second surgeries were not significantly related to patient age. During cataract surgery, the management of patient discomfort is important, not only for the patient's psychological well-being but also for influencing the surgical outcome. Therefore, the role of several factors that can influence patient comfort is a necessary additional consideration for surgeons treating patients who are undergoing consecutive surgeries for bilateral cataracts.

## Figures and Tables

**Table 1:** Patient demographic characteristics and concomitant diseases.

Characteristic	Number of patients (percent)
Gender	
Male	60 (47.2%)
Female	67 (52.8%)
Mean age ± SD (years)	69.8 ± 9.4
Concomitant diseases	
High myopia	38 (29.9%)
Hypertension	63 (49.6%)
Diabetes mellitus	30 (23.6%)
Heart disease	36 (28.3%)

**Table 2:** The number of patients of different age groups who reported changes in painful sensations between the first and second surgery.

Age (years)	First pain	Second pain	The same pain	No pain
<50	1	0	0	1
50–59	4	7	5	0
60–69	10	19	12	3
70–79	12	19	13	2
>79	6	8	5	0
*Sum*	*33*	*53*	*35*	*6*
Percent	26.0%	41.7%	27.6%	4.7%
